# Acupuncture Modulates the Cerebello-Thalamo-Cortical Circuit and Cognitive Brain Regions in Patients of Parkinson's Disease With Tremor

**DOI:** 10.3389/fnagi.2018.00206

**Published:** 2018-07-05

**Authors:** Zhe Li, Jun Chen, Jianbo Cheng, Sicong Huang, Yingyu Hu, Yijuan Wu, Guihua Li, Bo Liu, Xian Liu, Wenyuan Guo, Shuxuan Huang, Miaomiao Zhou, Xiang Chen, Yousheng Xiao, Chaojun Chen, Junbin Chen, Xiaodong Luo, Pingyi Xu

**Affiliations:** ^1^Department of Neurology, The First Affiliated Hospital, Guangzhou Medical University, Guangzhou, China; ^2^Department of Neurology, The Second Affiliated Hospital, Guangzhou University of Chinese Medicine, Guangzhou, China; ^3^Department of Radiology, The Second Affiliated Hospital, Guangzhou University of Chinese Medicine, Guangzhou, China; ^4^Department of Radiology, The People's Hospital of Gaozhou, Gaozhou, China; ^5^Department of Laboratory, The Second Affiliated Hospital, Guangzhou Medical University, Guangzhou, China; ^6^Department of Business Development, Zhujiang Hospital, Southern Medical University, Guangzhou, China; ^7^Department of Neurology, The First Affiliated Hospital, Sun Yat-sen University, Guangzhou, China; ^8^Department of Neurology, Guangzhou Hospital of Integrated Traditional and West Medicine, Guangzhou, China; ^9^Department of Neurology, Yuebei People's Hospital, Shaoguan, China

**Keywords:** acupuncture, Parkinson's disease, tremor, functional magnetic resonance imaging, neuromechanism

## Abstract

**Objective:** To investigate the effect of acupuncture on Parkinson's disease (PD) patients with tremor and its potential neuromechanism by functional magnetic resonance imaging (fMRI).

**Methods:** Forty-one PD patients with tremor were randomly assigned to true acupuncture group (TAG, *n* = 14), sham acupuncture group (SAG, *n* = 14) and waiting group (WG, *n* = 13). All patients received levodopa for 12 weeks. Patients in TAG were acupunctured on DU20, GB20, and the Chorea-Tremor Controlled Zone, and patients in SAG accepted sham acupuncture, while patients in WG received no acupuncture treatment until 12 weeks after the course was ended. The UPDRS II and III subscales, and fMRI scans of the patients' brains were obtained before and after the treatment course. UPDRS II and III scores were analyzed by SPSS, while the degree centrality (DC), regional homogeneity (ReHo) and amplitude low-frequency fluctuation (ALFF) were determined by REST.

**Results:** Acupuncture improved the UPDRS II and III scores in PD patients with tremor without placebo effect, only in tremor score. Acupuncture had specific effects on the cerebrocerebellar pathways as shown by the decreased DC and ReHo and increased ALFF values, and nonspecific effects on the spinocerebellar pathways as shown by the increased ReHo and ALFF values (*P* < 0.05, AlphaSim corrected). Increased ReHo values were observed within the thalamus and motor cortex of the PD patients (*P* < 0.05, AlphaSim corrected). In addition, the default mode network (DMN), visual areas and insula were activated by the acupuncture with increased DC, ReHo and/or ALFF, while the prefrontal cortex (PFC) presented a significant decrease in ReHo and ALFF values after acupuncture (*P* < 0.05, AlphaSim corrected).

**Conclusions:** The cerebellum, thalamus and motor cortex, which are connected to the cerebello-thalamo-cortical (CTC) circuit, were modulated by the acupuncture stimulation to alleviate the PD tremor. The regulation of neural activity within the cognitive brain regions (the DMN, visual areas, insula and PFC) together with CTC circuit may contributes to enhancing movement and improving patients' daily life activities.

## Introduction

Parkinson's disease (PD) is an age-related neurodegenerative disorder of unknown origin that is characterized by the selective loss of dopaminergic neurons in the substantia nigra pars compacta (Miller and O'Callaghan, 2015). Tremor is usually the first clinical sign of PD, and approximately 70% of PD patients manifest conspicuous tremor at rest and/or during the maintenance of posture (Wang, 2006). The management of PD tremor presents a number of challenges to clinicians (Jiménez and Vingerhoets, 2012). Medication, which is the first line of treatment, often has unpredictable side effects. Stereotactic surgery provides better clinical results than medication but is poorly accepted, due to its invasiveness and high cost (Jiménez and Vingerhoets, 2012). Thus, many physicians and patients desire a complementary alternative strategy for tremor management. Acupuncture is a promising traditional Chinese medicine therapy that can be used to treat PD, and ~7–10% of Asians choose acupuncture for tremor improvement (Lam et al., 2008). Due to its better adaptability, fewer side effects and lower cost, acupuncture has been widely used. Clinical studies have showed a positive benefit of acupuncture in treating PD tremor (Jiang et al., 2006; Wang et al., 2015). However, the mechanism underlying the effects of acupuncture on tremor associated with PD remains unknown.

Due to advances in brain neuroimaging technologies, recent functional magnetic resonance imaging (fMRI) studies have demonstrated that the basal ganglia (i.e., the pallidum and putamen) are active at the onset of tremor and the cerebellar circuit displays activity that is correlated with the magnitude of the ongoing tremor (Hallett, 2012). Both the basal ganglia and the cerebellum are connected to the motor cortex because the motor cortex is a component of both circuits, indicating the presence of pathology in the cerebello-thalamo-cortical (CTC) circuit in PD patients with tremor (Hallett, 2012).

fMRI is also a versatile tool to investigate the mechanism of acupuncture. According to previous animal studies, acupuncture plays a potential neuroprotective and restorative role in neuron survival (Kim et al., 2011; Sun et al., 2012; Rui et al., 2013; Xiao, 2015). This disease-modifying effect was reported to be similar to the effects of certain neuroprotective agents with anti-oxidative stress, anti-inflammatory and anti-apoptosis effects that improve motor performance in PD patients (Kim et al., 2011; Sun et al., 2012; Rui et al., 2013; Xiao, 2015). However, due to the physiological differences between humans and animals, conclusions based on animal experiments might differ from those based on clinical investigations involving human patients. fMRI can be used to visually measure the specific impact of acupuncture on the human brain (Deng et al., 2008, 2016; Zhang et al., 2012; Zhou et al., 2014; Zhang Q. et al., 2015; Zhang S. Q. et al., 2015). Although several fMRI studies have investigated the medical effect of acupuncture on PD symptoms, studies investigating tremor are limited (Chae et al., 2009; Su, 2009; Shang, 2010; Ye, 2011; Yeo et al., 2012, 2014). For example, Yeo et al. (2012, 2014) found that acupuncture stimulation on GB34 (Yanglingquan) activated substantia nigra, basal ganglia, precentral gyrus and prefrontal cortex in PD, but the authors were unable to determine the mechanism by which acupuncture decreased tremor.

Based on the abovementioned knowledge, we speculated that acupuncture might alleviate tremor and improve motor function in PD patients by modulating the CTC circuit or other pathways. Thus, here, we investigated the effectiveness of acupuncture paratherapy on PD patients with tremor and explore its underlying neuromechanism by fMRI analyzing the degree centrality (DC), regional homogeneity (ReHo), and amplitudes of low-frequency fluctuation (ALFF). The analytical processes used in these three methods are very similar and, thus, are useful for identifying regions with consistent activity across fMRI studies.

## Materials and methods

### Subjects

This study was conducted at the 2nd Affiliated Hospital of Guangzhou University of Chinese Medicine between May 2014 and January 2016. The patients included in this study were diagnosed based on the UK PD Society Brain Bank clinical diagnostic criteria, and tremor at rest in at least one upper or lower extremity on either side was assessed by item 20 of the Unified Parkinson's Disease Rating Scale (UPDRS) (Gibb and Lees, 1988; UKNCCF, 2006; Prodoehl et al., 2013). The exclusion criteria included secondary Parkinsonism, atypical parkinsonian disease, advanced PD stage (H-Y ≥ 4), age less than 45 or greater than 80 years, history of other neurological disorders or head trauma, left-handedness, cognitive impairment (Mini Mental State Examination (MMSE) score < 24), depression tendency (Beck Depression Inventory (BDI) score >4), and any contraindications for fMRI. The subjects were randomly assigned to a true acupuncture group (TAG), sham acupuncture group (SAG), or waiting group (WG) using a computer-generated list based on consecutive numbers that were distributed in sealed, opaque envelopes. All subjects provided written and verbal informed consent before participating in the study. They were informed what the study was about, including the possible risks and benefits to them, and were completely voluntary taking part in this study. They may also leave the study at any time. If they left the study before it was finished, there would be no penalty to them, and they would not lose any benefits to which they were otherwise entitled. This study was approved by the Ethics Committee of the 2nd Affiliated Hospital of Guangzhou University of Chinese Medicine.

### Acupuncture

All subjects in the three groups received conventional levodopa treatment for a course of 12 weeks. A single experienced acupuncturist, who was not blinded to the group assignment, performed acupuncture twice weekly. In the TAG, stainless steel needles were inserted to a depth of 2.0–3.0 cm into DU20 (Baihui), GB20 (Fengchi), and the Chorea-Tremor Controlled Zone to alleviate tremor according to traditional Chinese medicine documents. Chorea-Tremor Controlled Zone is located at the scalp above the front of precentral gurus, 1.5 cm before Motor Zone. The reinforcing-reducing method conducted by twirling was performed every 10 min within the 30-min needle retention time. In the SAG, needles were inserted to 0.2 cm deep and 0.5 Chinese cun next to DU20, GB20 and the Chorea-Tremor Controlled Zone, but no manipulation of the needle was performed during the needle retention time. In the WG, true acupuncture was performed for 12 weeks following the completion of the medication course and the acupuncture effect was not evaluated. To guarantee that the patients were blinded during the treatment period, patients in each group received acupuncture treatment in different independent single-rooms; and all patients received bilateral and equivalent number of acupoint each time.

### Clinical evaluation

Clinical evaluators, who remained blinded throughout the study, assessed the UPDRS II and III of all subjects before and after the treatment course. In UPDRS III section, items 20 and 21 are for the tremor score, 22 for the rigidity score, 23, 24, 25, 26, and 31 for the hypokinesia score, and 27, 28, 29, and 30 for the postural instability/gait disorder (PIGD) score (Liu et al., 2011). These four sub-scores represent the four typical motor symptoms of PD. Adverse effects of acupuncture were recorded if they happened.

### Image acquisition

The brains of all subjects were scanned using a 3.0 Tesla MRI (Siemens MAGNETOM Verio 3.0T, Erlangen, Germany) with an 8-channel phased-array head coil at the radiology department of the hospital before and after the treatment course. To eliminate the effect of levodopa on the brain, the fMRI scan was performed at least 4 h after the levodopa administration. During the data acquisition process, all subjects were asked to close their eyes and lie quietly for MR scanning. Resting-state functional images were acquired using a T2-weighted gradient-recalled echo-planar imaging (GRE-EPI) sequence with the following parameters: repetition time = 2,000 ms, echo time = 30 ms, flip angle = 90°, thickness = 3.5 mm, gap = 0.35 mm, field of view = 224 × 224 mm2, matrix = 64 × 64, 31 axial slices, and 240 time points. The structural images were analyzed using a three-dimensional T1-weighted magnetization-prepared rapid gradient echo (MPRAGE) sequence with the following parameters: repetition time = 1,900 ms, echo time = 2.27 ms, flip angle = 9°, thickness = 1.0 mm, field of view = 256 × 256 mm2, and matrix = 256 × 256.

### Data preprocessing and calculations

The fMRI data analyser was also blinded to the group assignment. The resting-state fMRI data preprocessing was performed using DPABI based on MATLAB (The Math Works, Natick, MA, USA). After removing the first four volumes of each participant, the functional images were corrected for the intra-volume acquisition time delay using slice-timing and realignment. None of the participants were excluded based on the criteria of displacement >2 mm or angular rotation >2° in any direction. All corrected functional data were then normalized to the Montreal Neurological Institute (MNI) space and resampled to a 3-mm isotropic resolution. The resulting images were further temporally band-pass filtered (0.01–0.08 Hz) to remove the effects of low-frequency drift and high-frequency physiological noise. Finally, 24 head-motion parameters, white matter signals, and cerebrospinal fluid signals were regressed using a general linear model, and linear trends were removed from the fMRI data. Spatial smoothing was also performed before the ALFF analysis using a Gaussian filter (6-mm full-width half-maximum, FWHM), but after the ReHo calculation.

REST (http://resting-fmri.sourceforge.net) (Song et al., 2011) V1.8 was used to calculate the values of the DC, ReHo, and ALFF. The DC represents the large-scale brain intrinsic connectivity related to the global information integration function at the voxel level (Buckner et al., 2009). We applied threshold to the correlation coefficients at *r* >0.25 to remove the weak correlations caused by noises. ReHo depicts the local synchronization of the time series of neighboring voxels, which is related to the local information integration function (Zang et al., 2004). ALFF measures the amplitude of time series fluctuations at each voxel and is thought to be associated with spontaneous neuronal activity (Zang et al., 2007). Thus, these three fMRI measures probe different aspects of brain activity (Wang et al., 2017).

### Statistical analysis

One-way ANOVA and the chi-squared test were performed to assess the baseline differences in the demographic and clinical data among the three groups, and paired-sample *t*-test was performed to evaluate UPDRS II and III scores before and after the treatment in each group using SPSS V22.0 (SPSS Inc., Chicago, IL, USA). The level of significance was set as *P* < 0.05.

The statistical analysis of the DC, ReHo and ALFF was conducted using REST V1.8. An ANCOVA was performed on the fMRI data to identify the DC, ReHo and ALFF maps among three groups with the pretreamtment images as covariates. Subsequently, the regions that showed significant differences were extracted as a mask, and the DC, ReHo, and ALFF values were subjected to post hoc analysis. Statistical comparisons of these values between each pair of groups were performed using a two-sample *post-hoc t*-test, and the LSD correction was applied for multiple tests to keep the overall type I error level of 0.05. Voxels with a *P* < 0.05 corrected by AlphaSim and a cluster size >2, 295 mm^3^ (85 voxels) were considered significantly different.

## Results

### Demographic and clinical characteristics

**Figure 1:**
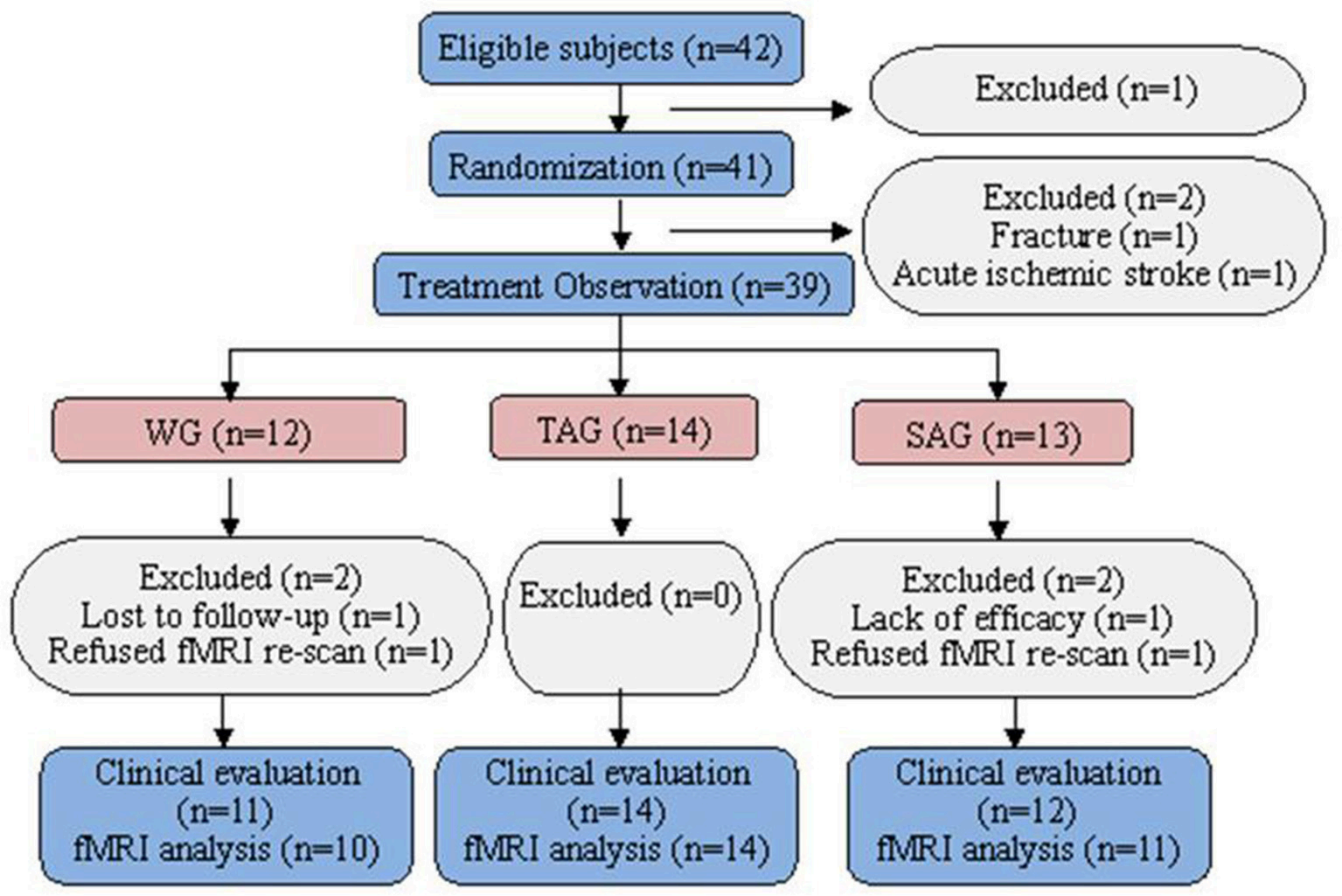
Flow chart of the enrolled subjects.

Of the 42 patients who were identified as potential participants, one patient refused the fMRI scanning. The remaining 41 patients were randomized in this study. No patients were withdrawn from the TAG. Two subjects, one in the SAG who was diagnosed with acute ischaemic stroke by fMRI and another in the WG who experienced an accidental fall, were excluded. Four subjects, 2 in the SAG and 2 in the WG, were withdrawn because of poor efficacy, failure to follow up or refusal to re-scan fMRI after the week 4 visit call (Figure 1). The baseline demographic and clinical characteristics of the patients in three groups are presented in Table 1. All subjects were cognitively normal and free of depression according to the MMSE and the BDI. No statistically significant differences in gender, age, family history, onset age, PD duration, UPDRS (II and III) or levodopa usage were observed among three groups.

**Table 1:** Baseline demographic and clinical data of all subjects.

	**WG (*n* = 12)**	**TAG (*n* = 14)**	**SAG (*n* = 13)**	***P*-value**
Gender (Male/Female)	9/3	8/6	7/6	0.51
Age (Years)	62.17 ± 7.66	65.79 ± 6.07	62.85 ± 5.00	0.30
Family history (Yes/No)	2/10	3/11	1/12	0.75
Onset age (Years)	54.83 ± 9.76	60.64 ± 7.56	57.85 ± 5.43	0.18
PD duration (Years)	7.33 ± 4.62	5.14 ± 3.32	5.03 ± 4.73	0.39
MMSE	27.17 ± 2.82	28.71 ± 1.20	28.69 ± 1.11	0.16
BDI	3.17 ± 1.34	2.36 ± 1.86	1.92 ± 1.66	0.17
UPDRS II score	11.42 ± 6.37	12.64 ± 5.98	11.38 ± 4.41	0.81
UPDRS III score	24.17 ± 13.44	26.00 ± 15.07	20.31 ± 8.64	0.58
Levodopa usage	345.83 ± 173.81	367.86 ± 146.24	338.46 ± 112.09	0.92

### Clinical evaluation

TAG showed significant improvement in UPDRS II and III scores, while WG and SAG didn't. In UPDRS III, tremor score in TAG decreased obviously while in WG increased significantly, but no big change in SAG. However, rigidity, hypokinesia and PIGD scores before and after treatment in each group displayed no significant differences (Table 2). No obvious adverse effects of acupuncture in the patients were reported.

**Table 2:** UPDRS II and III score of all subjects before and after treatment.

		**WG (*n* = 11)**	**TAG (*n* = 14)**	**SAG (*n* = 12)**
UPDRS II score	Pre-treatment	11.09 ± 6.58	12.64 ± 5.98	11.25 ± 4.58
	Post-treatment	12.27 ± 7.90	9.00 ± 4.40	10.33 ± 5.28
	T-value	−1.44	3.40	1.08
	*P*-value	0.179	0.005*	0.303
UPDRS III score	Pre-treatment	24.36 ± 14.07	26.00 ± 15.07	19.50 ± 8.49
	Post-treatment	26.73 ± 14.03	21.64 ± 13.68	19.25 ± 9.21
	T-value	−1.88	2.96	0.29
	*P*-value	0.090	0.011*	0.777
Tremor score	Pre-treatment	1.12 ± 0.62	1.17 ± 0.52	0.92 ± 0.49
	Post-treatment	1.31 ± 0.69	0.85 ± 0.43	0.90 ± 0.47
	T-value	−3.75	6.19	0.19
	*P*-value	0.004*	0.000*	0.851
Rigidity score	Pre-treatment	0.71 ± 0.72	0.89 ± 0.82	0.52 ± 0.54
	Post-treatment	0.80 ± 0.64	0.76 ± 0.79	0.50 ± 0.53
	T-value	−1.46	1.26	0.43
	*P*-value	0.176	0.229	0.674
Hypokinesia score	Pre-treatment	0.85 ± 0.53	0.84 ± 0.75	0.56 ± 0.32
	Post-treatment	0.91 ± 0.52	0.71 ± 0.77	0.58 ± 0.30
	T-value	−0.69	1.85	−0.39
	*P*-value	0.506	0.087	0.701
PIGD score	Pre-treatment	0.82 ± 0.66	0.88 ± 0.45	0.79 ± 0.58
	Post-treatment	0.82 ± 0.63	0.82 ± 0.36	0.75 ± 0.62
	T-value	0.00	0.90	0.69
	*P*-value	1.000	0.385	0.504

Data are shown as mean ± SD. *P < 0.05.

### DC

An ANCOVA revealed significant differences in the DC index between the TAG, SAG, and WG in the following regions: fusiform gyrus, cuneus, lingual gyrus, superior and middle occipital gyri, insula and cerebellum crus. Compared with the SAG, the TAG showed increased DC in the fusiform gyrus, cuneus, lingual gyrus, superior and middle occipital gyri, and decreases in the cerebellum crus. Compared with the WG, the TAG displayed increased DC in the cuneus and decreased DC in the cerebellum crus. In addition, compared with the WG, the SAG's DC values were significantly elevated in bilateral insula. The details of the peak coordinates and cluster sizes are listed in Table 3.

**Table 3:** Brain regions exhibiting increased and decreased degree centrality among three groups.

**Brain regions**	**Cluster size**	**Peak intensity**	**Peak MNI coordinates**		
			**X**	**Y**	**Z**
**TAG**>**SAG**					
Right Fusiform Gyrus	100	4.68	33	−48	−15
Bilateral Cuneus, Lingual Gyrus, Superior and Middle Occipital Gyri, Fusiform Gyrus	1,181	6.28	−12	−90	15
**TAG**<**SAG**					
Left Cerebellum Crus1 and 2	97	−3.96	−36	−78	−27
**TAG**>**WG**					
Bilateral Cuneus	116	4.12	3	−81	36
**TAG**<**WG**					
Left Cerebellum Crus1 and 2	131	−5.30	−39	−84	−33
**SAG**>**WG**					
Left Insula	183	4.42	−27	9	12
Right Insula	110	4.65	42	−9	12

*TAG, true acupuncture group; SAG, sham acupuncture group; WG, waiting group. A > B, Compared with B group, A group showed increased DC values; A < B, Compared with B group, A group showed decreased DC values (P < 0.05, AlphaSim corrected)*.

### ReHo

**Figure 2:**
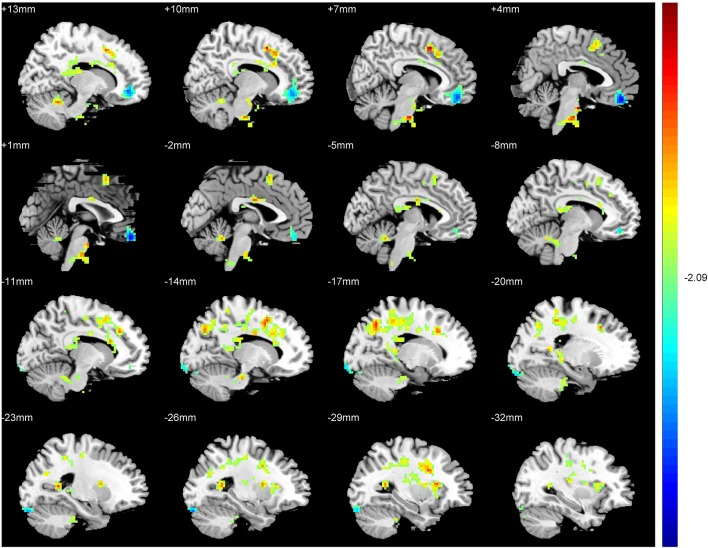
Differences in ReHo values between the TAG and SAG. (*P* < 0.05, AlphaSim corrected). Warm colors represent positive ReHo values; blue (cold) colors represent negative ReHo values.

**Figure 3:**
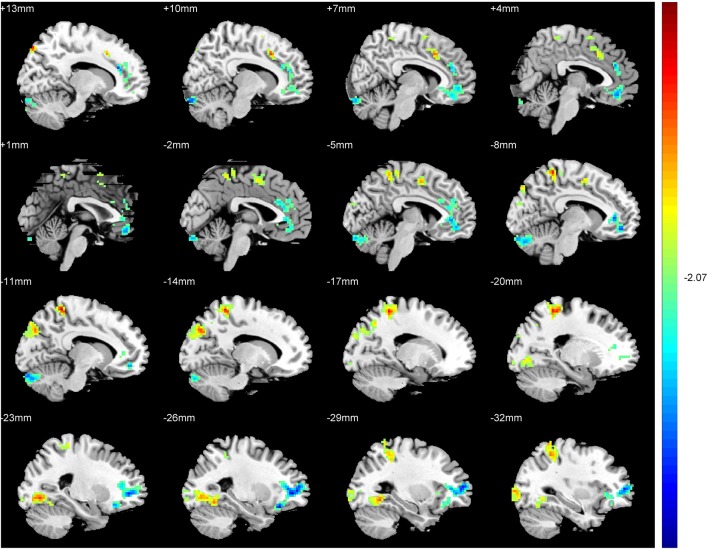
Differences in ReHo values between the TAG and WG. (*P* < 0.05, AlphaSim corrected). Warm colors represent positive ReHo values; blue (cold) colors represent negative ReHo values.

An ANCOVA exhibits significant differences in the ReHo index among three groups in the following regions: middle and inferior frontal gyri, rectus gyrus, precentral gyrus, supplement motor area (SMA), inferior parietal lobules, precuneus, cuneus, fusiform gyrus, superior and middle cccipital gyri, anterior cingulate gyrus, hippocampus, thalamus, insula and cerebellum. Compared with the SAG, a significantly increased ReHo values was detected in the SMA, inferior parietal lobules, precuneus, cuneus, fusiform gyrus, superior and middle cccipital gyri, and a significant decrease in the ReHo was observed in the middle and inferior frontal gyri, anterior cingulate gyrus and the cerebellum crus of patients in the TAG (Figure 2). Compared to the WG, patients of the TAG had an enhanced ReHo values in the left precentral gyrus, SMA, precuneus, hippocampus, thalamus, insula and cerebellum 4_5, and a reduced ReHo values in the middle and inferior frontal gyri, rectus gyrus, right precentral gyrus and cerebellum crus (Figure 3). What's more, the SAG patients displayed an increased ReHo in the left insula and a decreased regional activity in the right precentral gyrus compared to the WG. The details of the peak coordinates and cluster sizes are listed in Table 4.

**Table 4:** Brain regions exhibiting increased and decreased regional homogeneity among three groups.

**Brain regions**	**Cluster size**	**Peak intensity**	**Peak MNI coordinates**		
			**X**	**Y**	**Z**
**TAG>SAG**					
Left Fusiform Gyrus	144	4.13	−24	−75	−6
Left Middle Occipital Gyrus	127	3.96	−39	−93	−3
Left Cuneus and Superior Occipital Gyrus	123	3.98	−15	−78	36
Right Cuneus and Superior Occipital Gyrus	89	5.36	15	−84	48
Bilateral Supplement Motor Area	108	4.18	9	15	39
Left Precuneus	198	5.12	−12	−45	63
Left Inferior Parietal Lobules	91	3.68	−33	−48	51
**TAG<SAG**					
Bilateral Cerebellum Crus1 and 2	181	−3.99	9	−93	−24
Left Middle and Inferior Frontal Gyri	360	−5.00	−27	27	−18
Bilateral Anterior Cingulate Gyrus	566	−4.57	15	33	18
**TAG>WG**					
Right Cerebellum_4_5	264	5.10	3	−12	−33
Left Precuneus, Hippocampus and Thalamus	189	4.31	−24	−51	9
Right Hippocampus and Precuneus	377	4.55	18	−45	15
Bilateral Supplement Motor Area	747	5.51	6	9	48
Left Precentral Gyrus	97	4.29	−30	6	30
Left Insula	141	4.14	−30	18	12
**TAG<WG**					
Right Rectus and Middle Frontal Gyrus	416	−4.77	3	48	−21
Left Cerebellum Crus1	89	−4.04	−27	−90	−24
Right Orbital Inferior Frontal Gyrus	95	−3.95	42	42	−15
Right Precentral Gyrus	98	−5.47	54	0	42
**SAG>WG**					
Left Insula	107	4.42	−27	9	12
**SAG<WG**					
Right Precentral Gyrus	162	−5.70	51	0	42

*TAG, true acupuncture group; SAG, sham acupuncture group; WG, waiting group. A > B, Compared with B group, A group showed increased ReHo values; A < B, Compared with B group, A group showed decreased ReHo values (P < 0.05, AlphaSim corrected)*.

### ALFF

The inferior and medial frontal gyri, fusiform gyrus and the cerebellum revealed significant differences in the ALFF values among the TAG, SAG and WG. Compared to the SAG, an enhanced ALFF in the cerebellum 4_5 and 6, and a reduced ALFF in the orbital inferior frontal gyrus were observed in the patients of TAG. Furthermore, compared to the WG, significantly elevated spontaneous neural activities exhibited in the fusiform gyrus, cerebellum crus and vermis of the TAG patients, and in the medial frontal gyrus and cerebellum crus of the SAG patients. The details of the peak coordinates and cluster sizes are listed in Table 5.

**Table 5:** Brain regions exhibiting increased and decreased amplitude of low frequency fluctuations among three groups.

**Brain regions**	**Cluster size**	**Peak intensity**	**Peak MNI coordinates**		
			**X**	**Y**	**Z**
**TAG**>**SAG**					
Left Cerebellum 4_5 and 6	94	3.95	−36	−33	−27
**TAG**<**SAG**					
Right Orbital Inferior Frontal Gyrus	217	−4.24	27	36	−12
**TAG**>**WG**					
Right Fusiform Gyrus	396	4.54	48	−36	−27
Left Fusiform Gyrus	320	4.82	−33	−33	−27
Right Cerebellum_Crus1	176	3.25	33	−57	0
Left Cerebellum_Crus1	273	2.94	−39	−66	−33
Vermis_4_5 and 6	167	3.54	3	−57	−15
**SAG**>**WG**					
Right Medial Frontal Gyrus	93	3.89	15	42	12
Right Cerebellum_Crus1	339	4.38	18	−81	−27

*TAG, true acupuncture group; SAG, sham acupuncture group; WG, waiting group; A > B, Compared with B group, A group showed increased ALFF values; A < B, Compared with B group, A group showed decreased ALFF values (P < 0.05, AlphaSim corrected)*.

## Discussion

In this neural imaging study, we investigated the effect and its neural substrates of acupuncture stimulation on PD patients with tremor by the DC, ReHo and ALFF methods. We proved that acupuncture could improve the daily life activities and motor symptoms in PD patients with tremor without placebo effect, and tremor was the only symptom that had been ameliorated in the four typical motor symptoms of PD. We then analyzed the fMRI data that the DC, ReHo and ALFF analyses were consistent in the spinocerebellum, thalamus, default mode network (DMN), insula, visual areas and prefrontal cortex (PFC), and inconsistent in the cerebrocerebellum and motor cortex.

Interestingly, the acupuncture administration exerted a specific activating effect on the cerebrocerebellum (cerebellum crus1 and 2, and cerebellum 6) with decreased DC and ReHo values and enhanced ALFF signals, and a nonspecific effect on the spinocerebellum (cerebellum 4_5 and vermis) associated with the enhanced ReHo and ALFF values. A significant change of the ReHo value in the thalamus and motor cortex (precentral gyrus and SMA) was also observed. Anatomically, the cerebrocerebellum receives input neural signals from the cerebral cortex and sends output signals mainly to ventrolateral thalamus in turn connected to motor areas of the premotor cortex and the primary motor area of the cerebral cortex, which is thought to be involved in the initiation, planning and coordination of movements (Kandel and Schwartz, 1985; Schmahmann et al., 1999). The conflicting signal changes in the cerebrocerebellum may be due to a compensation by the activated left precentral gyrus and SMA for the function of the cerebellum in integrating information with the motor cortex. The inconsistent ReHo changes in the left and right precentral gyri could be attributed to the fact that all subjects in our study were right-handed; in these patients, the improved function of the left precentral gyrus might be expected to compensate for the decreased function of the right precentral gyrus. The connections of the cerebellum, thalamus and motor cortex to the CTC circuit have been shown to influence patients' functional motor activity (Wu and Hallett, 2013). Thus, our data demonstrate acupuncture improve the motor function and daily life activities of PD patients by a direct stimulatory effect on the CTC circuit. Moreover, several imaging studies have suggested that PD tremor is strongly associated with the CTC circuit (Helmich et al., 2011, 2012; Zhang J. et al., 2015), which may be the reason that tremor was the only motor symptoms being improved. Although this study may be the first time to elucidate how acupuncture affects PD tremor, evidence for tremor control via the CTC network has been accumulated by deep brain stimulation (DBS) and repetitive transcranial magnetic stimulation (rTMS), and several reports indicate that the stimulating mechanism of DBS or rTMS is potentially similar to that of acupuncture (Fukuda et al., 2004; Mure et al., 2011; Popa et al., 2013; Coenen et al., 2014).

We also observed that acupuncture had a specific effect on brain regions relevant to cognitive activity, such as the DMN (the anterior cingulate gyrus, precuneus, cuneus, medial PFC, inferior parietal lobule and hippocampus), visual areas (the lingual gyrus, superior and middle occipital gyri and the fusiform gyrus), insula and PFC (gyrus rectus, middle and inferior frontal gyri). The effect of DMN and insula on cognitive processing has been confirmed in recent fMRI studies investigating aging individuals and individuals with neurodegenerative disorders (Tessitore et al., 2012; Zhang S. Q. et al., 2015; Jiang et al., 2016). The visual processing controlled by the visual areas and the executive function managed by the PFC (Yuan and Raz, 2014) are also parts of the cognition. Since the subjects enrolled in this study were cognitively normal, these cognitive brain regions is speculated to participate in the cognitive management of movement, as movement control includes motor and cognitive components (Prevosto and Sommer, 2013). Generally, the cognitive control of movement is achieved by motion perception, movement learning, movement memory, movement planning and motor inhibition (Lu et al., 2012). For instance, in humans, an environmental stimulus related to motion is first perceived by the visual system, and the produced visual information is conveyed from the visual areas to the motor cortex to generate motion perception. Subsequently, an empirical rule is formed from the learning and memory of movement, which results from neural activity in the primary motor cortex, cerebellum, DMN and insula. Then, movement planning is regulated by the basal ganglia, SMA and PFC to enable the execution of precise action (Lu et al., 2012). Therefore, the modulation of the neural activity in cognitive regions may contribute to the movement improvement along with the CTC circuit.

Several limitations of this study should be addressed. First, this was a small sample study, and the authors did not continue to assess the clinical symptoms and fMRI after the treatment course was discontinued. Second, there was 6 drop-outs out of 41 randomized patients (15%), which might cause bias in the conclusion. Third, we used MMSE to screen for the cognitive impairments as it's quite specific and easy to complete. Nonetheless, MMSE may not be as sensitive as the Montreal Cognitive Assessment to detect mild cognitive impairments. A study with a larger patient population, long-term follow-up and reliable cognitive tests is needed to more conclusively determine the efficacy and its neuromechanism of acupuncture on PD tremor and find out whether acupuncture could work on the cognition for a longer period of time by modulating the cognitive brain regions.

In conclusion, our findings reveal that acupuncture has specific and nonspecific effects on different brain regions involved in PD tremor, and the motor and cognitive management of movement. The underlying mechanism of the effects of acupuncture on PD tremor may be related to a modification of the CTC circuit, and the modulation of the cognitive functional regions together with CTC circuit contributes to enhancing movement and improving the daily life activities of PD patients.

## Author contributions

ZL, BL, and XDL conceived and designed the experiments. JBC and XL performed the fMRI scans. JC analyzed the fMRI data. GHL performed the acupuncture. WYG and YSX analyzed the clinical data. SCH, SXH, MMZ, and XC collected the clinical data. YJW, CJC, JBC, and XDL recruited potential participants. and ZL, YYH, and PYX wrote the manuscript. All authors read and approved the final manuscript.

### Conflict of interest statement

The authors declare that the research was conducted in the absence of any commercial or financial relationships that could be construed as a potential conflict of interest.
